# Are the Spinal Changes in the Course of Scoliogeny Primary but Secondary?

**DOI:** 10.3390/jcm13082163

**Published:** 2024-04-09

**Authors:** Theodoros B. Grivas, Elias Vasiliadis, Christina Mazioti, Despina Papagianni, Aristea Mamzeri, Michail Chandrinos, George Vynichakis, Konstantinos Athanasopoulos, Paschalis Christodoulides, Nikola Jevtic, Samra Pjanic, Danka Ljubojevic, Olga Savvidou, Angelos Kaspiris, Jarrett Grunstein

**Affiliations:** 1 Trauma and Orthopaedic Department, Former Head, “Tzaneio” General Hospital of Piraeus, 185 36 Piraeus, Greece; 2 3rd Department of Orthopaedics, School of Medicine, National and Kapodistrian University of Athens, KAT Hospital, 145 61 Athens, Greece; eliasvasiliadis@yahoo.gr (E.V.); angkaspiris@hotmail.com (A.K.); 3 “Tzaneio” General Hospital of Piraeus, 185 36 Piraeus, Greece; maziotix@gmail.com; 4 School Nurse, Special Primary School of Rafina, 190 09 Rafina, Greece; papdes2009@hotmail.com; 5 TOMY Attica Square, 104 45 Athens, Greece; mamzeri_aristea@hotmail.com; 6 Orthopedic Department, Gen. Hospital of Argolida-N.M. Argous, 212 00 Argos, Greece; chandrinosmichail@gmail.com (M.C.); vini_gio@windowslive.com (G.V.); 7 Orthopedic Department, Peristeri Medical Group, 121 32 Peristeri, Greece; drathkon@yahoo.gr; 8 Department of Radiology, General Hospital of Paphos, Paphos 8026, Cyprus; pasxalis.xris@gmail.com; 9 Scolio Centar, 403916 Novi Sad, Serbia; njevticns@gmail.com (N.J.); danka.ljubojevic93@gmail.com (D.L.); 10 Department of Paediatric Rehabilitation, Institute for Physical, Rehabilitation Medicine and Orthopaedic Surgery “Dr Miroslav Zotovic”, 78000 Banja Luka, Bosnia and Herzegovina; samra.pjanic@hotmail.com; 11 First Department of Orthopaedic Surgery, School of Medicine, National and Kapodistrian University of Athens, “ATTIKON” University General Hospital, Rimini 1, 124 62 Athens, Greece; nikoleta02@yahoo.com; 12 Laboratory of Molecular Pharmacology, Department of Pharmacy, School of Health Sciences, University of Patras, 265 04 Patras, Greece; 13 Chiropractic Center Livingston, 340 E Northfield Rd # 2E, Livingston, NJ 07039, USA; jgspineez@gmail.com

**Keywords:** idiopathic scoliosis, vertebral spine, rib cage, genetics, epigenetics, sleep

## Abstract

In this opinion article, there is an analysis and discussion regarding the effects of growth on the spinal and rib cage deformities, the role of the rib cage in scoliogeny, the lateral spinal profile in adolescent idiopathic scoliosis (AIS), the genetics and epigenetics of AIS, and the interesting and novel field investigating the sleep impact at nighttime on AIS in relation to the sequence of the scoliogenetic changes in scoliotics. The expressed opinions are mainly based on the published peer-reviewed research of the author and his team of co-authors. Based on the analysis noted above, it can be postulated that the vertebral growth changes in the spine during initial idiopathic scoliosis (IS) development are not primary-intrinsic but secondary changes. The primary cause starting the deformity is not located within the vertebral bodies. Instead, the deformations seen in the vertebral bodies are the secondary effects of asymmetrical loads exerted upon them, due to muscular loads, growth, and gravity.

## 1. Introduction

An unanswered question in the scoliogeny is whether the growth changes in the spine in initial idiopathic scoliosis development and mild similar cases are primary/inherent or secondary. There is no clear answer, and there is limited information on this issue in the literature. Some state that pathology begins within the spine [1], while others argue that changes in the spine are secondary [2]. The research approach to shed some light on this issue is multidimensional, with the study of the various anatomical components of the deformity, such as the thoracic cage, the lateral spinal profile, the intervertebral discs, the genetics and epigenetics in idiopathic scoliosis, and the impact of sleep period of time on idiopathic scoliosis.

It was reported that in the rib cage in adolescent idiopathic scoliosis, the rib length asymmetry in the apical region is a secondary event to the scoliosis deformity and not a protagonistic feature in the aetiopathogenesis [3,4]. The opponents of this view claim that in the chain of pathological deformations leading to scoliosis, the ribs deform first and then the spine follows [5,6].

In humans, it is a recognized axiom that anatomy and physiology are interdependent, as one determines the other. In an attempt to answer the question “Are the scoliogenetic changes in the spine primary or secondary?” it will be helpful and suitable to use this formerly described concept as the morphology–anatomy expresses–reflects and deciphers–decodes the physiology and pathology and vice versa.

The knowledge of normality is necessary for the study of abnormality. The proper way to study the mechanisms of a deformity development is when this is initiating and mild or even moderate and not when it is progressed. The question arises as to how to find sufferers from mild or even moderate forms of idiopathic scoliosis, as well as a normal peer group population. The answer may be found by analyzing the children involved in the school scoliosis screening program, which, beyond its original aim, which is prevention, is a human evidence-based clinical research tool of IS scoliogeny established on the concept mentioned above, which is as follows: morphology expresses–reflects and deciphers–decodes the physiology and pathology. This school scoliosis screening program will help to find sufferers from mild, or even moderate, forms of scoliosis and at the same time identify a plethora of normal peers for comparison.

During the twenty-five years of school scoliosis screening program implementation, from 1997 to 2021, 24.223 school children and adolescents, 5–18 years of age, were examined and the collected data was analyzed. A special feature of our program was that a wide age range of children was examined, which is a strategy that was not reported to be applied in other school scoliosis screening programs. The analysis of the thesaurus of all these data resulted in some very interesting findings. It was discovered that the younger children with truncal asymmetry who were referred to the scoliosis clinic often times had a perfectly straight spine with no vertebral rotation, despite the presence of a thoracic hump [7]. The scoliotic spine first deforms at the level of the intervertebral disc, not the vertebrae [8,9].

The thoracic cage develops asymmetrically first; consequently, we suggested the “Double Rib Contour Sign” in the lateral radiographs [10], and the “rib index” [11,12], for the assessment of the thoracic cage deformity in the transverse plane. The lateral spinal profile of mild idiopathic scoliosis of 10°–20° Cobb angle, is not a predisposing factor for the initiation of IS curves; it is rather a compensatory mechanism [13].

The analysis and discussion of the effect of growth on spinal and rib cage deformity, of intervertebral discs in adolescent idiopathic scoliosis, of the role of the thorax in scoliogeny, of the lateral spinal profile in adolescent idiopathic scoliosis, and the genetics and epigenetics and the interesting “new” field of study of the sleep impact at night-time on adolescent idiopathic scoliosis are presented in this opinion report in relation to the sequence of the scoliogenetic changes in scoliotics.

## 2. Effect of Growth on Spinal and Rib Cage Deformity

It was discovered that the younger children with truncal asymmetry who were referred to the scoliosis clinic often times had a perfectly straight spine with no vertebral rotation, despite the presence of a thoracic hump. Approximately 30% of younger referred SSS girls, less than 13 years of age with an angle of trunk rotation equal to or more than seven degrees, were found to have either a straight spine or a spinal curve with a Cobb angle less than 10 degrees. In this age group, the correlation between clinical deformity in terms of truncal/thoracic asymmetry assessed using the rib index [12] and radiographic measurement, in terms of Cobb angle, is not statistically significant, while in older school scoliosis screening referred girls, aged 14–18 yrs. old, it is [7]. Furthermore, it was reported that whilst adolescent idiopathic scoliosis features both vertebral body rotation and torso asymmetry, they are poorly related to each other [14].

## 3. Intervertebral Disc and Adolescent Idiopathic Scoliosis

The study of the radiographical assessment of our referred school scoliosis screening children suffering mild IS showed that the deformity starts at the level of the intervertebral disc, then the vertebra body, as a result of the plasticity of the intervertebral disc [8,9]. Three years later, this finding was confirmed in another report [15]. In pertinent published research, histological abnormalities in the intervertebral discs of adolescent idiopathic scoliosis cases were discovered, and it was concluded that these abnormalities are secondary to a changed mechanical environment [16,17,18,19,20].

It was shown using computer tomography technology that, for the anterior length of the spine, the intervertebral discs contribute more compared to the vertebral body [21]. It was also supported that this finding suggests that the curve is getting worse due to changed mechanical loading and not a primary vertebral growth abnormality [21].

**Figure 1:**
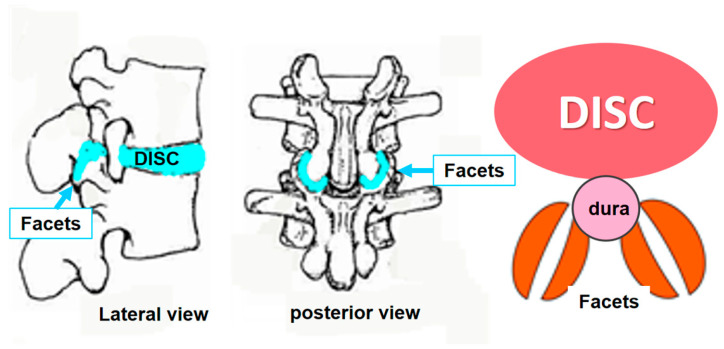
The three-joint complex.

As far as the role of the IVDs with scoliogeny, a comprehensive model of IS progression, based on the patho-biomechanics of the deforming “three joint complex” was presented [22]. The “three joint complex” concept was introduced by Dr. WH Kirkadly Willis in 1983. A “three-joint complex” was coined the intervertebral disc anteriorly and the two facet joints posteriorly in the intervertebral articulation [23], Figure 1.

It is suggested that the patho-biomechanics of the deforming “three joint complex” are due to asymmetrical concentrated cyclical loads to the apical and adjacent immature vertebral end plates and posterior elements of the spine due to water diurnal variation in intervertebral disc leading to asymmetrical vertebral growth.

**Figure 2:**
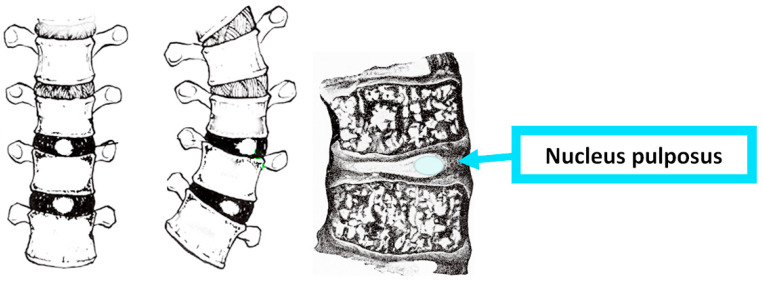
The nucleus pulposus (NP—green color) in IS migrates to the convex side.

In idiopathic scoliosis, the intervertebral disc is very deformed. At the concavity of the idiopathic scoliosis curve, the intervertebral disc height is significantly reduced. On the contrary, at the convexity, the intervertebral disc height is increased. Consequently, much of the deformity of idiopathic scoliosis belongs to this alteration in the intervertebral disc [24]. In idiopathic scoliosis, the nucleus pulposus, mainly at the apical but progressively less so at the adjacent intervertebral discs, is shifted to the convexity of the curve [25], Figure 2, and it is also reported that the worsening of IS is related to this phenomenon [26,27].

Aggrecan, a proteoglycan that aggregates by binding to hyaluronan, is one of the nucleus pulposus molecules. Glycosaminoglycans are attached to each aggrecan molecule [28]. Glycosaminoglycans in the nucleus pulposus function osmotically, causing a change in the amount of intervertebral disc water. The glycosaminoglycans imbibe water when they are unloaded and expel it when the intervertebral disc is loaded. The swelling of the intervertebral disc correlates directly with glycosaminoglycan content [29,30,31].

Human height varies throughout the 24 h period, lengthening when a subject lies down and shortening while in a standing position. This is called diurnal variation [32]. The diurnal variation phenomenon relates to the spine. This diurnal variation was reported to be due to fluctuation in the water content of the intervertebral disc [33,34]. Glycosaminoglycans imbibe water when the intervertebral discs are unloaded during sleep at night and expel the water when they are loaded in the upright position during the day. In idiopathic scoliosis, the imbibed water mainly in the apical but also in the neighboring intervertebral discs of the curve is more on the convex side than on the concave side due to convex-wise asymmetrical distribution of glycosaminoglycans in the nucleus pulposus collagen network type II.

This diurnal variation and asymmetrical amount of water distribution in the apical and adjacent intervertebral discs results in asymmetrical, convex-wise, concentrated cyclical loads to the intervertebral disc during the 24 h cycle. The convex side of the wedged intervertebral disc sustains a greater amount of expansion, due to nucleus pulposus swelling, than the concave side.

**Figure 3:**
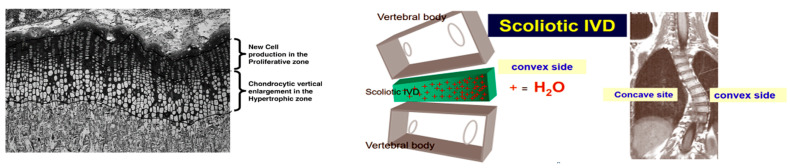
The endplates on the convex vertebral side have an increasing rate of proliferation of chondrocytes in their hypertrophic zone.

On the convex vertebral side due to water diurnal variation, intermittent forces are transmitted to the developing vertebral endplates of chondrocytes in the hypertrophic zone according to the laws of Pauwels and Wolff. The concave side of the curve is practically continuously loaded with compressive forces and the growth slows down accordingly in the hypertrophic zone of the endplates according to the Hueter–Volkmann law, Figure 3. Consequently, the imposed convex-wise, asymmetrically concentrated cyclical loads on the adjacent immature vertebral end plates lead to asymmetrical further vertebral body growth, as an effect of Pauwels law. As far as the posterior elements of the spine, the loading on the two facet joints asymmetrically increases during the day, as the wedged disc space narrows due to expelled water, and it asymmetrically decreases during the night, because the disc space swells due to reabsorbed water; consequently, these posterior elements of the spine grow asymmetrically too. It is well described that the pedicles are different between the concave and the convex side, as well as the facet joint and the lamina [35,36,37].

This suggested comprehensive model of IS progression describes the vertebral body deformation that, however, assumes the wedge-shaped deformation of the intervertebral disc a fact that occurs first before any deformation of the vertebra in scoliogenesis, as it was shown [8].

**Figure 4:**
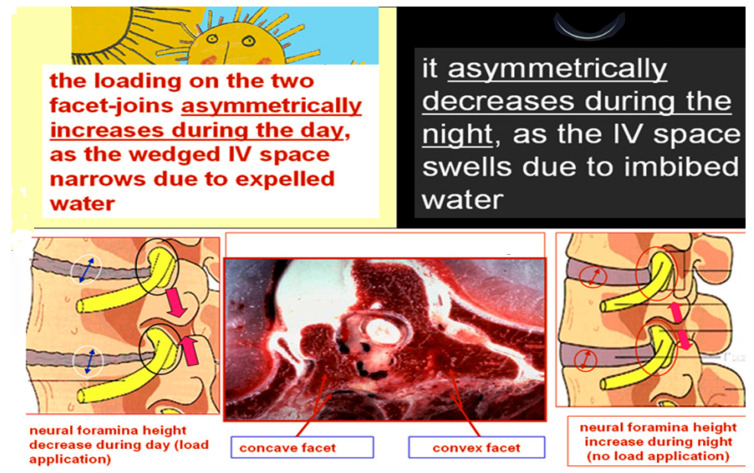
The diurnal variation accordion-like phenomenon during day and night [38].

The above-described patho-mechanism of spinal progression in idiopathic scoliosis has been coined the “accordion-like phenomenon” [38], Figure 4. The comprehensive model of idiopathic scoliosis progression, based on the patho-biomechanics of the deforming “three joint complex”, may help to explain the beneficial effects of physiotherapeutic scoliosis specific exercise bracing and of fusion-less surgery for progressive early onset scoliosis [22].

## 4. The Role of the Thoracic Cage in IS Scoliogeny [39]

The normal rib cage includes the thoracic spine, thoracic spinal cord, heart, lungs, diaphragm, respiratory muscles, and sternum. The rib cage’s main function is in respiration as it acts as a respiratory pump and protects the enclosed organs. Similarly, the ribs support the thoracic vertebrae. They also serve as levers through which the forces exerted by the attached muscles and ligaments to the vertebrae through their costovertebral articulations [40].

**Figure 5:**
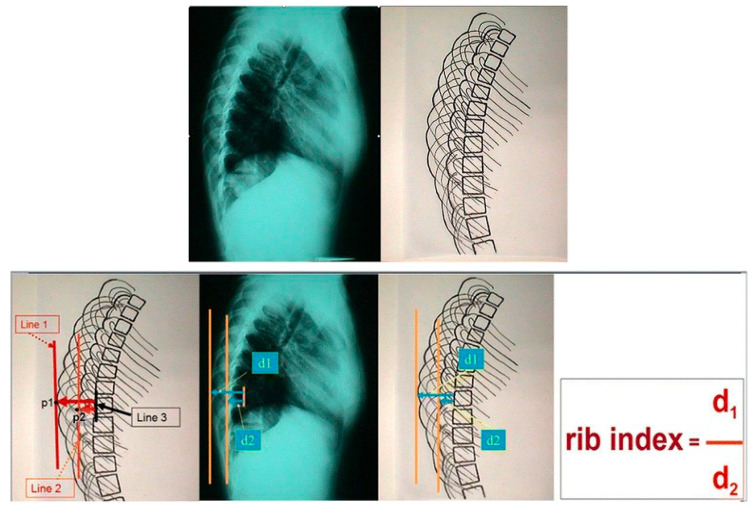
The DRCS and the RI, ref. [12].

In idiopathic scoliosis, the rib cage seems to develop asymmetrically first before the spinal deformation. Consequently, we suggested the “double rib contour sign” in the standing lateral full-body radiographs and introduced the “rib index” for the assessment of the thoracic cage deformity in the transverse plane [10,12]. In the standing lateral full-body radiographs of all asymmetric children with a rib hump deformity, the radiologic sign of a double rib contour, which was termed “double rib contour sign”, is seen [10]. The quantification of the “rib index” in a lateral radiograph is measured following the steps as earlier reported [12], see also Figure 5: the rib index is the quotient of d1/d2.

## 5. Segmental Rib Index, Spinal Deformity, and the Scoliogenic Implications [41]

**Figure 6:**
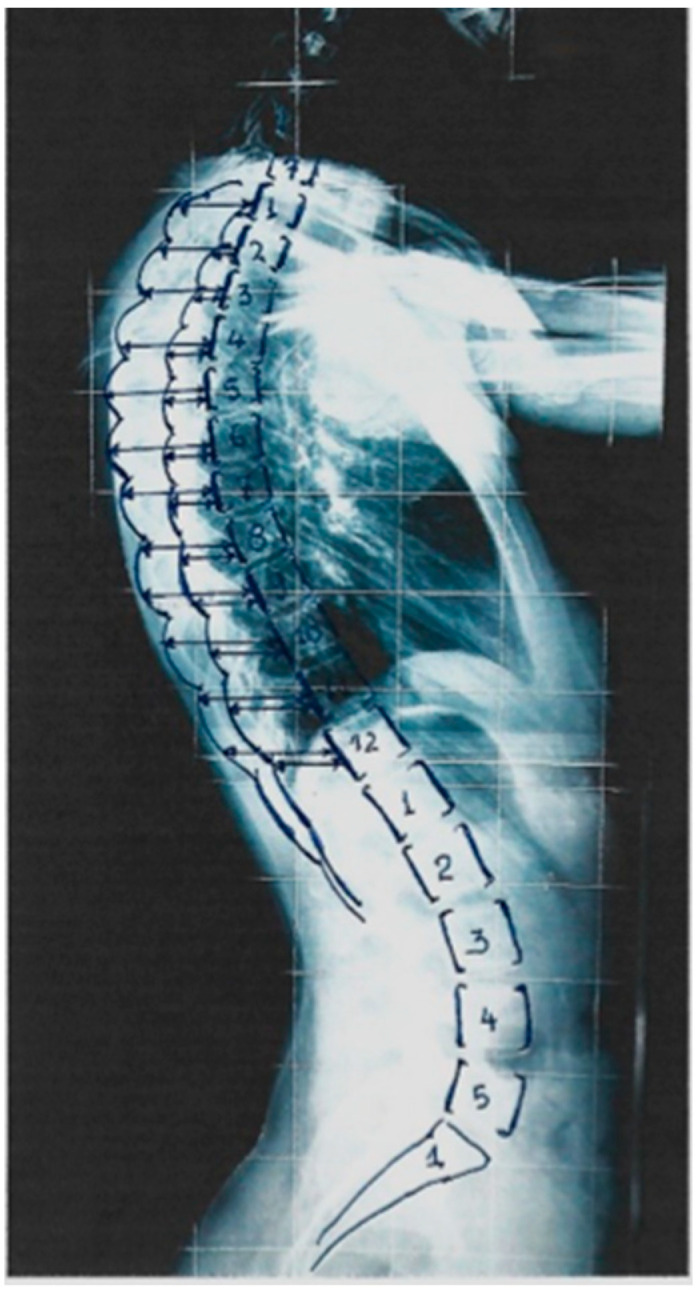
The way the segmental rib index (T1–T12) is assessed in the lateral spinal standing radiographs.

The rib index calculation was originally implemented at the apex of the double rib contour sign in the lateral standing spinal radiographs. We noticed that in mild and moderate idiopathic scoliosis lateral spinal standing radiographs, the more prominent/distant point of the double rib contour sign is at a variable thoracic vertebral level in the different types of idiopathic scoliosis. Therefore, we studied the rib index segmentally at all thoracic vertebral levels (T1–T12) to evaluate the association of the Cobb angle of the type of the idiopathic scoliosis curve with the thoracic level of the deformity of the rib hump. The segmental rib index is calculated the same way at the upper and lower thoracic levels from T1 toT12, Figure 6 [41].

In mild and moderate idiopathic scoliosis curves, the vertebral rotation is minimal. Thus, rib cage deformity can generally be attributed to the asymmetric rib growth and to their deformation and not to the vertebral rotation, as the rotation at this stage is minimal. Therefore, at any level from T1 to T12, a value of segmental RI equal to or greater than 1.45–1.50 mainly reflects a significantly asymmetrical double rib contour, which is a fact indicating a remarkable asymmetrical growth of a pair of ribs at this spinal level. Therefore, this value of RI represents an increasing and progressive rib cage deformity. Consequently, the assessment of the correlation of the 12 rib pairs’ rib index to the spinal deformity helps to validate the impact of thoracic asymmetry on the spinal deformity and on its curve type.

The term “pattern of segmental rib index asymmetry” is used to indicate the number of rib levels, from T1 to T12, with a high rib index score that is equal to or greater than 1.45–1.50. In female patients with thoracic curves, the pattern of segmental rib index asymmetry was present in eight levels from T3 to T10, and in male patients with thoracolumbar curves, remarkably, a significant pattern of segmental rib index asymmetry was present in nine levels, from T3 to T5 and T7 to T12. Additionally, comparing the segmental rib index of the asymmetric but not scoliotic children to the scoliotic peers by curve type for boys and girls, interestingly, no significant difference between groups was found (non-scoliotic to thoracic, non-scoliotic to thoracolumbar, non-scoliotic to lumbar) [41]. These findings suggest that in mild and moderate idiopathic scoliosis, the link of the surface with radiological deformity shows the significant impact of the rib cage on the spinal deformity. The rib cage seems to play a protagonistic role in the scoliogeny of mild and moderate thoracic and thoracolumbar idiopathic scoliosis.

These findings are in line with the findings published earlier. It was stated that hump size was found to be the most powerful predictor of scoliosis [42]. Large humps were more prevalent among those children that subsequently developed IS. The predictive significance of baseline truncal asymmetry was independent of all the other determinants entered in the multifactorial logistic model, (sitting height, kyphosis, lordosis, arm length inequality, pelvic tilt). Boys with humps of 6 mm had approximately a fivefold risk of developing IS as compared with boys having a symmetric trunk (hump = 0 mm) at the age of 10.8 years. Additionally, it was reported that the asymmetric children with a hump deformity, but without radiographically diagnosed scoliosis, during a follow-up of three years, will develop IS with an odds ratio of 1.72 in boys and 1.55 in girls [43]. It was also reported that, on the moiré photographs with the children standing in the erect position, 12% of the girls and 9% of the boys with clinically observed asymmetries in the forward bending position had very small shadow asymmetries, using the moiré topography method. Also, in former Malmo studies, these small asymmetries of the trunk were not related to a lateral deviation of the spine, seen roentgenographically, exceeding nine degrees of Cobb angle [44,45,46].

As far as the lumbar curves were concerned, it was found that there was asymmetrical growth of the 12th rib pair. The following hypotheses were suggested: (a) relatively increased activity of the right quadratus lumborum muscle, which is a muscle that is related both to respiration and gait, causes the lateral lumbar curves (first hypothesis); or, (b) it counteracts the lumbar curvature as part of the body’s attempt to compensate for the curvature (second hypothesis). It was also suggested that mechanotransduction leads to a relatively increased length of the right 12th rib in accordance with Wolff’s and Pauwel’s Laws, ref. [47].

A review of the literature dealing with the postoperative correction of rib hump after spinal operations for adolescent idiopathic scoliosis shows that surgery on the spine cannot correct the asymmetry of the ribs of the rib cage or stop the mechanism that causes their asymmetrical growth in idiopathic scoliosis. Not only is the hump incompletely corrected but it recurs and worsens during the follow-up and even more intensively in skeletally immature operated scoliosis children. These results presented in all the relevant reviewed articles support the important protagonistic role of rib hump deformity on scoliogenesis, which precedes the subsequent formed spinal deformity [48], as was also previously noted in this opinion article [7]. Characteristically, Lofti et al., 2020, stated that “vertebral body rotation and torso asymmetry are poorly related to each other” and “surgical de-rotation of the spine does not fully address the rib hump as other factors”. Additionally, these authors “raised the question of whether or not current surgical methods involving de-rotation of the vertebral column actually address the problem that can affect the patient–namely the thoracic rib hump. If surgery is going to reduce the size of the rib hump through spinal de-rotation, then it would be important to demonstrate that vertebral body rotation is associated with thorax asymmetry and rotation” [14].

Using the segmental rib vertebra angle [49], and the segmental thoracic ratios methods [50], in posteroanterior thoracic radiographs both of early- and of late-onset scoliotic children with mild curves, it was shown that they have an underdeveloped thoracic cage compared to nonscoliotic counterparts [51,52,53,54].

It was suggested the hypothesis that rib vertebral angles are influenced by the central nervous system through its influence on trunk muscle activity. Rib vertebra angle asymmetries or rib vertebral angle differences are related to age and sex; their pattern reflects the common age, sex, and laterality patterns of idiopathic scoliosis [49]. Additionally, as the rib cage role in idiopathic scoliosis scoliogeny, it was suggested that extremes of such asymmetries may be an aetiological factor for both infantile and adolescent IS [49].

The authors’ opinion on the role of the rib cage in the IS scoliogeny is in line with Prof. Sevastik’s research work, pertinent to scoliosis aetiology. These reports emphasize the important role of the rib cage in scoliosis development and support a physiological approach to the surgical treatment of progressive early idiopathic scoliosis [55,56,57,58,59,60,61,62,63,64,65,66,67]. Segmental rib index research may likewise shed more light on the theory of asymmetric function of the autonomous nervous system, reported by Prof. Sevastik and his team [5].

## 6. Lateral Spinal Profile and Adolescent Idiopathic Scoliosis

While studying the lateral spinal profile, in school screening referrals with and without late-onset idiopathic scoliosis with mild curves 10°–20° Cobb angle, it was shown that it is not statistically different in children with straight spines, with spinal curvature having a Cobb angle less than 10° and children with thoracic, thoracolumbar, and lumbar curves of 10°–20° [13]. These findings on lateral spinal profiles relate to aetiological importance in idiopathic scoliosis pathobio-mechanics and show that in mild idiopathic scoliosis, hypokyphosis is not a predisposing factor for the initiation of these curves, as it is not different from the lateral spinal profile of their healthy controls. The lateral spinal profile is rather a compensatory than an aetiological factor for scoliogeny [13]. Therefore, hypokyphosis is not a primary causal factor for the commencing of mild or moderate scoliotic curves, but it could be considered a permissive factor in the scoliogeny of AIS. These findings also indicate that the growth potential in the sagittal plane in mild and moderate IS is similar to that of peers having normal spines, in both vertebral bodies and the intervertebral discs [13,68].

## 7. Genetics and Epigenetics

Recent research has focused on idiopathic scoliosis-related genes. Several types of pathogenic gene mutations have been identified in idiopathic scoliosis through genetic studies; however, no single gene responsible for idiopathic scoliosis has been found so far [69,70,71,72,73].

A significant body of research indicates the presence of diverse morphological features, clinical presentations, and prognoses among adolescent idiopathic scoliosis patients. Complexity and heterogeneity are key characteristics of adolescent idiopathic scoliosis aetiology and phenotype, suggesting that adolescent idiopathic scoliosis can be considered a relatively complex group of diseases [74,75,76]. Consequently, it is clear and reported in the literature that several genes are involved in the creation of this deformity. This raises the question of whether adolescent idiopathic scoliosis deformity/disease is a single one or multiple diseases.

When discussing adolescent idiopathic scoliosis genetics, it is very important to include some more details on epigenetics related to this deformity. Epigenetics is generally accepted now to play a significant role in the formation of the final phenotype; see several pertinent epigenetic factors and reasons reported in pertinent publications on the role of epigenetics:

- Monozygotic twins and spinal radiology in adolescent idiopathic scoliosis [77,78,79,80,81,82,83,84,85,86,87,88,89,90,91];
- A food and growth connection [92,93,94,95,96,97,98,99,100,101,102,103,104];
- Relative osteopenia and lifestyle factors [75,105,106];
- Physical activities of patients with adolescent idiopathic scoliosis [107,108,109];
- Geographic latitude and the prevalence of adolescent idiopathic scoliosis [110,111];
- Maternal age and socio-economic status [112,113,114,115,116,117];
- Heated indoor swimming pools infants and delayed epigenetic effects [108,109,118];
- Hypothesis of developmental instability for scoliosis [99,101,119,120,121];
- Sleeping period of time and sleeping position [38,122].

The genetic and epigenetic factors creating the various types of idiopathic scoliosis phenotype seem to be due to a “long” spectrum of causes. The one end of this spectrum seems to be the rib cage, while the other end of the spectrum seems to be the vertebral column. Therefore, the authors’ opinions on the patho-biomechanics of mild and moderate IS support the “rib cage end” of the spectrum of genetic and epigenetic factors responsible for initial ribcage asymmetry as triggering the idiopathic scoliosis deformity, rather than the “spine end” of these factors of the spectrum.

Viroli et al. [123] report “the presence of a more severe pedicle dysplasia in the proximal, nonstructural, thoracic curves compared to the main, structural, thoracic curves”. There is no doubt that pedicle dysplasia is a crucial aspect nowadays since the widespread of all-pedicle-screw constructs for posterior scoliosis correction. However, we declare in our report that “the proper way to study the mechanisms of a deformity development is when this is initiating and mild or even moderate and not when it is progressed”. Our opinion was based on the study of initiating mild and moderated scoliosis cases and not progressing. In this interesting article [123], the studied cohort of patients is preoperative, in other words, very much progressed scoliosis. Therefore, the population is not comparable with ours on which our opinion was based.

The used methods of imaging, the ethics based on describing the requested imaging, their reliability study, the ages of children, and the degree of deformity are analyzed in detail in our earlier publications, refs. [7,8,9,10,12,13,22,38,41,47,48,49,50,51,52,110].

A limitation of the described opinion of this report may be considered that the thoracic and spinal data segmental thoracic ratios [50], rib vertebral angle and rib vertebral angle differences [124], segmental rib vertebral angle [49], rib index [11,12] and segmental rib index [41], double rib couture sign [10], and a lateral spinal profile study [13]), which were used at the first author’s peer review publications cited in the literature, were based on radiographs that provide two-dimensional imaging and information. However, recently, the assessment of the thoracic and spinal deformity evaluation by the use of 3-D evaluation methods [125,126,127,128,129] certainly offers interesting possibilities, but it requires special equipment. From a practical point of view, the studies based on postero-anterior radiographs may have a valuable contribution. The most important and frequently used radiological parameters are designed and measured on postero-anterior radiographs (Mehta [124], Cobb [130], Perdriolle [131,132,133], Nash Moe grade of rotation [134]). Lateral radiographs are not systematically made for children with scoliosis. In the majority of hospitals, the material accessible for retrospective studies contains almost exclusively frontal plane radiographs. Moreover, the plain chest radiographs of children and adolescents, being easily available at medical archives, can effectively serve this study, without the need for any other special radiographs and exposure to additional radiation. One additional benefit of these methods is their implementation, not only in prospective but also in retrospective studies, using the existing initially obtained chest or spinal radiographs of IS patients, provided that the radiography is performed in a standard way.

Future research based on the clinical and imaging long-term follow-up of children and adolescents, screened at school and found with asymmetric rib index but without scoliosis, may be very helpful as they will confirm, support, and maybe refine this reported opinion. Additionally, the knowledge of the driving scoliogenetic risk factors for severe idiopathic scoliosis based on rib cage deformity will be very helpful for their management.

## 8. Conclusions

Based on the analysis presented above, it can be postulated that the vertebral growth changes in the spine during initial IS development are not primary-intrinsic but secondary changes. The primary cause starting the deformity is not based on the vertebral bodies, which subsequently deform due to asymmetrical loads exerted upon them, due to muscular loads, growth, and gravity.

## References

[1] Zhu F., Chu W.C.W., Sun G., Zhu Z.Z., Wang W.J., Cheng J.C.Y., Qiu Y. (2011). Rib length asymmetry in thoracic adolescent idiopathic scoliosis: Is it primary or secondary?. Eur. Spine J..

[2] Burwell R.G., Cole A.A., Cook T.A., Grivas T.B., Kiel A.W., Moulton A., Thirlwall A.S., Upadhyay S.S., Webb J.K., Wemyss-Holden S.A. (1992). Pathogenesis of idiopathic scoliosis. The Nottingham concept. Acta Orthop. Belg..

[3] Qiu Y., Sun G.Q., Zhu F., Wang W.J., Zhu Z.Z. (2010). Rib length discrepancy in patients with adolescent idiopathic scoliosis. Stud. Health Technol. Inform..

[4] Schlager B., Krump F., Boettinger J., Jonas R., Liebsch C., Ruf M., Beer M., Wilke H.J. (2021). Morphological patterns of the rib cage and lung in the healthy and adolescent idiopathic scoliosis. J. Anat..

[5] Sevastik J.A. (2002). Dysfunction of the autonomic nerve system (ANS) in the aetiopathogenesis of adolescent idiopathic scoliosis. Stud. Health Technol. Inform..

[6] Sevastik J., Burwell R.G., Dangerfield P.H. (2003). A new concept for the etiopathogenesis of the thoracospinal deformity of idiopathic scoliosis: Summary of an electronic focus group debate of the IBSE. Eur. Spine J..

[7] Grivas T.B., Vasiliadis E.S., Mihas C., Savvidou O. (2007). The effect of growth on the correlation between the spinal and rib cage deformity: Implications on idiopathic scoliosis pathogenesis. Scoliosis.

[8] Grivas T.B., Vasiliadis E., Malakasis M., Mouzakis V., Segos D. (2006). Intervertebral disc biomechanics in the pathogenesis of idiopathic scoliosis. Stud. Health Technol. Inform..

[9] Grivas T.B., Vasiliadis E.S., Rodopoulos G., Bardakos N. (2008). The role of the intervertebral disc in correction of scoliotic curves. A theoretical model of idiopathic scoliosis pathogenesis. Stud. Health Technol. Inform..

[10] Grivas T.B., Daggas S., Polyzois B., Samelis P. (2002). The double rib contour sign in lateral spinal radiographs. Aetiologic implications for scoliosis?. Stud. Health Technol. Inf..

[11] Grivas T.B., Dangas S., Lafogianni S., Samelis P., Polyzois D. The Double Rib Contour Sign (DRCS) in lateral spinal radiographs: Aetiologic implications for scoliosis. Proceedings of the 25th “Nicolas Giannestras—Panayiotis Smyrnis” Anniversary Symposium of Spinal Column Diseases.

[12] Grivas T.B. (2014). Rib index. Scoliosis.

[13] Grivas T.B., Daggas S., Samelis P., Cmazioti C., Kandris P. (2002). Lateral spinal profile in school-screening referrals with and without late onset idiopathic scoliosis 10°–20°. Stud. Health Technol. Inform..

[14] Lotfi N., Chauhan G.S., Gardner A., Berryman F., Pynsent P. (2020). The relationship between measures of spinal deformity and measures of thoracic trunk rotation. J. Spine Surg..

[15] Will R.E., Stokes I.A., Qiu X. (2009). Cobb angle progression in adolescent scoliosis begins at the intervertebral disc. Spine.

[16] Colten H.R., Altevogt B.M. (2006). Sleep Disorders and Sleep Deprivation: An Unmet Public Health Problem.

[17] Shu C.C., Melrose J. (2018). The adolescent idiopathic scoliotic IVD displays advanced aggrecanolysis and a glycosaminoglycan composition similar to that of aged human and ovine (sheep) IVDs. Eur. Spine J..

[18] Antoniou J., Arlet V., Goswami T., Aebi M., Alini M. (2001). Elevated synthetic activity in the convex side of scoliotic intervertebral discs and endplates compared with normal tissues. Spine.

[19] Roberts S., Menage J., Eisenstein S.M. (1993). The cartilage end-plate and intervertebral disc in scoliosis: Calcification and other sequelae. J. Orthop. Res..

[20] Grivas T.B., Vasiliadis E.S., Kaspiris A., Khaldi L., Kletsas D. (2011). Expression of matrix metalloproteinase-1 (MMP-1) in Wistar rat’s intervertebral disc after experimentally induced scoliotic deformity. Scoliosis.

[21] Brink R.C., Schlösser T.P.C., van Stralen M., Vincken K.L., Kruyt M.C., Hui S.C.N., Viergever M.A., Chu W.C.W., Cheng J.C.Y., Castelein R.M. (2018). Anterior-posterior length discrepancy of the spinal column in adolescent idiopathic scoliosis-a 3D CT study. Spine J..

[22] Grivas T.B., Vasiliadis E.S., Triantafyllopoulos G., Kaspiris A. (2009). A comprehensive model of idiopathic scoliosis (IS) progression, based on the patho-biomechanics of the deforming “three joint complex. Scoliosis.

[23] Yong-Hing K., Kirkaldy-Willis W.H. (1983). The pathophysiology of degenerative disease of the lumbar spine. Orthop. Clin. N. Am..

[24] Taylor T.F.K., Ghosh P., Bushell G.R. (1981). The contribution of the intervertebral disk to the scoliotic deformity. Clin. Orthop..

[25] O’Brien J., Siegler D., Harrison D., Edgar M. (1988). Mechanisms of progression in neuromuscular scoliosis. Proceedings of the Eighth Philip Zorab Scoliosis Symposium.

[26] Toyama Y. (1988). An experimental study on the pathology and role of intervertebral discs in the progression and correction of scoliotic deformity. Nippon Seikeigeka Gakkai Zasshi.

[27] Perie D., Curnier D., de Gauzy J.S. (2003). Correlation between nucleus zone migration within scoliotic intervertebral discs and mechanical properties distribution within scoliotic vertebrae. Magn. Reson. Imaging.

[28] Jobnstone Β., Bayliss Μ.T., Aspden R.M., Porter Κ.W. (1995). The extracellular matrix of the intervertebral disc: Proteoglycan biochemistry. Lumbar Spine Disorders: Current Concepts.

[29] Urban J.P., Maroudas A., Bayliss M.T., Dillon J. (1979). Swelling pressures of proteoglycans at the concentrations found in cartilaginous tissues. Biorheology.

[30] Urban J.P.G., McMullin J.F., Jacobs R.R. (1984). The relationship between disc proteoglycan content and disc height. Pathogenesis of Scoliosis. Proceedings of an International Conference.

[31] Urban J.P., Maroudas A. (1981). Swelling of the intervertebral disc in vitro. Conn. Tiss. Res..

[32] Dangerfield P., Roberts N., Walker J., Betal D., Edwards R.H.T., D’Amico M., Merolli A., Santambrogio G.C. (1995). Investigation of the diurnal variation in the water content of the intervertebral disc using MRI and its implications for scoliosis. The Three Dimensional Analysis of Spinal Deformities.

[33] De Puky P. (1935). The Physiological oscillation of the length of the body. Acta Orthop. Scand..

[34] Czaprowski D., Tyrakowski M., Bloda J., Waś J., Dembińska A., Ewertowska P., Kotwicki T. (2019). Diurnal variation of body height in children with idiopathic scoliosis. J. Back Musculoskelet. Rehabil..

[35] Gao B., Gao W., Chen C., Wang Q., Lin S., Xu C., Huang D., Su P. (2017). What is the Difference in Morphologic Features of the Thoracic Pedicle Between Patients with Adolescent Idiopathic Scoliosis and Healthy Subjects? A CT-based Case-control Study. Clin. Orthop. Relat. Res..

[36] Hu X., Siemionow K.B., Lieberman I.H. (2014). Thoracic and lumbar vertebrae morphology in Lenke type 1 female adolescent idiopathic scoliosis patients. Int. J. Spine Surg..

[37] Sakti Y.M., Lanodiyu Z.A., Ichsantyaridha M., Wijanarko S., Filza M.R., Taufan T., Susanto D.B., Tampubolon Y.O., Baskara A.A.N.N., Nurshal A.A. (2023). Pedicle morphometry analysis of main thoracic apex adolescent idiopathic scoliosis. BMC Surg..

[38] Grivas T.B. (2021). The Diurnal Variation “accordion”-like Phenomenon of Wedged Intervertebral Discs: A Progression Factor in Idiopathic Scoliosis. Ann. Pediatr. Child. Health.

[39] Grivas T.B. (2023). Thorax and Idiopathic Scoliosis. Int. J. Adv. Res..

[40] Bisgard J.D. (1934). Thoracogenic scoliosis: Influence of thoracic disease and thoracic operation on the spine. Arch. Surg..

[41] Grivas T.B., Jevtic N., Ljubojevic D., Pjanic S., Golic F., Vasiliadis E. (2023). Segmental Rib Index and Spinal Deformity: Scoliogenic Implications. Healthcare.

[42] Nissinen M., Heliovaara M., Tallroth K., Poussa M. (1989). Trunk asymmetry and scoliosis. Anthropometric measurements in prepubertal school children. Acta Paediatr. Scand..

[43] Nissinen M., Heliovaara M., Seltsamo J., Poussa M. (1993). Trunk Asymmetry, Posture, Growth, and Risk of Scoliosis. A Three-Year Follow-Up of Finnish Prepubertal School Children. Spine.

[44] Willner S. (1984). Development of trunk asymmetries and structural scoliosis in prepubertal school children in Malmo: Follow up study of children 10-14 years of age. J. Pediatr. Orthop..

[45] Willner S. (1979). Moiré topography-A method for school screening of scoliosis. Arch. Orthrop. Traumat. Surg..

[46] Willner S. (1983). The efficiency of a combined clinical- moiré technique in school screening of scoliosis. Moir Fringe Topography.

[47] Grivas T.B., Burwell R.G., Kechagias V., Mazioti C., Fountas A., Kolovou D., Christodoulou E. (2016). Idiopathic and normal lateral lumbar curves: Muscle effects interpreted by 12th rib length asymmetry with pathomechanic implications for lumbar idiopathic scoliosis. Scoliosis Spinal Disord..

[48] Grivas T.B., Vasiliadis E., Vynichakis G., Chandrinos M., Athanasopoulos K., Christodoulides P. (2023). Why Is There Always a Remnant Rib Hump Deformity after Spinal Operations in Idiopathic Scoliosis: Aetiological Implications and Recognition of the Proper Rib Level for Costoplasty. Children.

[49] Grivas T.B., Burwell R.G., Purdue M., Webb J.K., Moulton A. (1992). Segmental patterns of rib-vertebra angles in chest radiographs of children: Changes related to rib level, age, sex, side and significance for scoliosis. Clin. Anat..

[50] Grivas T.B., Burwell R.G., Purdue M., Webb J.K. (1991). A segmental analysis of thoracic shape in chest radiographs of children. Changes related to spinal level age sex side and significance for scoliosis. J. Anat..

[51] Grivas T.B., Samelis P., Chadziargiropoulos T., Polyzois B. (2002). Study of the rib cage deformity in children with 10 degrees-20 degrees of Cobb angle late onset idiopathic scoliosis, using rib-vertebra angles--aetiologic implications. Stud. Health Technol. Inform..

[52] Grivas T.B., Burwell G.R., Vasiliadis E.S., Webb J.K. (2006). Scoliosis. A segmental radiological study of the spine and rib—Cage in children with progressive infantile idiopathic scoliosis. Scoliosis.

[53] Grivas T.B., Burwell R.G., Purdue M., Webb J.K., Moulton A., Alberty A., Drerup B., Hierholzer E. (1992). The rib-cage deformity in infantile idiopathic scoliosis-the funnel-shaped upper chest in relation to specific rotation as a prognostic factor. An evaluation of thoracic shape in progressive scoliosis and control children during growth. Surface Topography and Spinal Deformity VI.

[54] Grivas T.B., Burwell R.G., Webb J.K. (1991). The funnel-shaped upper chest of progressive infantile idiopathic scoliosis (IIS): Significance for rib growth patterns, rib dysplasia and aetiology of the spinal deformity. Clin. Anat..

[55] Sevastik J.A., Aaro S., Normelli H. (1984). Scoliosis. Experimental and clinical studies. Clin. Orthop. Relat. Res..

[56] Normelli H., Sevastik J., Wallberg H. (1986). The thermal emission from the skin and the vascularity of the breasts in normal and scoliotic girls. Spine.

[57] Sevastik J.A., Aaro S., Lindholm S.T., Dalhborn M. (1987). Experimental scoliosis in growing rabbits by operations on the rib cage. Clin. Orthop..

[58] Agadir M., Sevastik B., Sevastik J.A., Persson A., Isberg B. (1988). Induction of scoliosis in the growing rabbit by unilateral rib-growth stimulation. Spine.

[59] Normelli H., Sevastik J.A., Ljung G., Jönsson-Söderström A.M. (1986). The symmetry of the breasts in normal and scoliotic girls. Spine.

[60] Sevastik J., Agadir M., Sevastik B. (1990). Effects of rib elongation on the spine: I. Distortion of the vertebral alignment in the rabbit. Spine.

[61] Sevastik J., Agadir M., Sevastik B. (1990). Effects of rib elongation on the spine: II. Correction of scoliosis in the rabbit. Spine.

[62] Agadir M., Sevastik B., Reinholt F.P., Perbeck L., Sevastik J. (1990). Vascular Changes in the Chest Wall After Unilateral Resection of the Intercostal Nerves in the Growing Rabbit. J. Orthop. Res..

[63] Sevastik B., Xiong B., Lundberg A., Sevastik J.A. (1995). In vitro opto-electronic analysis of 3-D segmental vertebral movements during gradual rib lengthening in the pig. Acta Orthop. Belg..

[64] Gréalou L., Aubin C.E., Sevastik J.A., Labelle H. (2002). Simulations of rib cage surgery for the management of scoliotic deformities. Stud. Health Technol. Inform..

[65] Normelli H., Sevastik J., Ljung G., Aaro S., Jönsson-Söderström A.M. (1985). Anthropometric data relating to normal and scoliotic Scandinavian girls. Spine.

[66] Sevastik J.A. (2006). Right convex thoracic female adolescent scoliosis in the light of the thoracospinal concept. Stud. Health Technol. Inform..

[67] Xiong B., Sevastik J.A. (1998). A physiological approach to surgical treatment of progressive early idiopathic scoliosis. Eur. Spine J..

[68] Grivas T.B., Vynichakis G., Chandrinos M., Mazioti C., Papagianni D., Mamzeri A., Mihas C. (2021). Morphology, Development and Deformation of the Spine in Mild and Moderate Scoliosis: Are Changes in the Spine Primary or Secondary?. J. Clin. Med..

[69] Marie-Hardy L., Courtin T., Pascal-Moussellard H., Zakine S., Brice A. (2023). The Whole-Exome Sequencing of a Cohort of 19 Families with Adolescent Idiopathic Scoliosis (AIS): Candidate Pathways. Genes.

[70] Lau K.K.L., Law K.K.P., Kwan K.Y.H., Cheung J.P.Y., Cheung K.M.C. (2023). Proprioception-related gene mutations in relation to the aetiopathogenesis of idiopathic scoliosis: A scoping review. J. Orthop. Res..

[71] AlMekkawi A.K., Caruso J.P., El Ahmadieh T.Y., Palmisciano P., Aljardali M.W., Derian A.G., Al Tamimi M., Bagley C.A., Aoun S.G. (2023). Single Nucleotide Polymorphisms and Adolescent Idiopathic Scoliosis: A Systematic Review and Meta-Analysis of the Literature. Spine.

[72] Nada D., Julien C., Papillon-Cavanagh S., Majewski J., Elbakry M., Elremaly W., Samuels M.E., Moreau A. (2022). Identification of FAT3 as a new candidate gene for adolescent idiopathic scoliosis. Sci. Rep..

[73] De Salvatore S., Ruzzini L., Longo U.G., Marino M., Greco A., Piergentili I., Costici P.F., Denaro V. (2022). Exploring the association between specific genes and the onset of idiopathic scoliosis: A systematic review. BMC Med. Genom..

[74] Ru L., Zheng H., Lian W., Zhao S., Fan Q. (2023). Knowledge mapping of idiopathic scoliosis genes and research hotspots (2002–2022): A bibliometric analysis. Front. Pediatr..

[75] Wang W.J., Yeung H.Y., Chu W.C.W., Tang N.L.S., Lee K.M., Qiu Y., Burwell R.G., Cheng J.C.Y. (2011). Top Theories for the Etiopathogenesis of Adolescent Idiopathic Scoliosis. J. Pediatr. Orthop. J. Pediatr. Orthop..

[76] Liu G., Wang L., Wang X., Yan Z., Yang X., Lin M., Liu S., Zuo Y., Niu Y., Zhao S. (2019). Whole-Genome Methylation Analysis of Phenotype Discordant Monozygotic Twins Reveals Novel Epigenetic Perturbation Contributing to the Pathogenesis of Adolescent Idiopathic Scoliosis. Front. Bioeng. Biotechnol..

[77] Wynne-Davies R. (1968). Familial (idiopathic) scoliosis. A family survey. J. Bone Jt. Surg. Br..

[78] Smyrnis T., Antoniou D., Valavanis J., Zachariou C. (1987). Idiopathic scoliosis: Characteristics and epidemiology. Orthopedics.

[79] Kesling K.L., Reinker K.A. (1997). Scoliosis in twins. A meta-analysis of the literature and report of six cases. Spine.

[80] Inoue M., Minami S., Kitahara H., Otsuka Y., Nakata Y., Takaso M., Moriya H. (1998). Idiopathic scoliosis in twins studied by DNA fingerprinting: The incidence and type of scoliosis. J. Bone Jt. Surg. Br..

[81] Van Rhijn L.W., Jansen E.J., Plasmans C.M., Veraart B.E. (2001). Curve characteristics in monozygotic twins with adolescent idiopathic scoliosis: 3 new twin pairs and a review of the literature. Acta Orthop. Scand..

[82] Fraga M.F., Ballestar E., Paz M.F., Ropero S., Setien F., Ballestar M.L., Heine-Suñer D., Cigudosa J.C., Urioste M., Benitez J. (2005). Epigenetic differences arise during the lifetime of monozygotic twins. Proc. Natl. Acad. Sci. USA.

[83] Martin G.M. (2005). Epigenetic drift in aging identical twins. Proc. Natl. Acad. Sci. USA.

[84] Andersen M.O., Thomsen K., Kyvik K.O. (2007). Adolescent idiopathic scoliosis in twins: A population-based survey. Spine.

[85] Hermus J.P., van Rhijn L.W., van Ooij A. (2007). Non-genetic expression of adolescent idiopathic scoliosis: A case report and review of the literature. Eur. Spine J..

[86] Miller N.H. (2007). Genetics of familial idiopathic scoliosis. Clin. Orthop. Relat. Res..

[87] Kaspiris A., Grivas T.B., Weiss H.R. (2008). Congenital scoliosis in monozygotic twins: Case report and review of possible factors contributing to its development. Scoliosis.

[88] Sales de Gauzy J., Ballouhey Q., Arnaud C., Grandjean H., Accadbled F. (2010). Concordance for curve type in familial idiopathic scoliosis: A survey of one hundred families. Spine.

[89] Wong C.C., Caspi A., Williams B., Craig I.W., Houts R., Ambler A., Moffitt T.E., Mill J. (2010). A longitudinal study of epigenetic variation in twins. Epigenetics.

[90] Grauers A., Rahman I., Gerdhem P. (2010). Heritability of scoliosis in the SwedishTwin Registry. Stud. Health Technol. Inform..

[91] Day J.J., Sweatt J.D. (2011). Epigenetic mechanisms in cognition. Neuron.

[92] Golding J. (1991). Observations on idiopathic scoliosis: Aetiology and natural history in Jamaica—A food and growth connection. Cajanus.

[93] Worthington V., Shambaugh P. (1993). Nutrition as an environmental factor in the etiology of idiopathic scoliosis. J. Manip. Physiol. Ther..

[94] Enwonwu C.O., Sanders C. (2001). Nutrition: Impact on oral and systemic health. Compend. Contin. Educ. Dent..

[95] Kaati G., Bygren L.O., Pembrey M., Sjöström M. (2007). Transgenerational response to nutrition, early life circumstances and longevity. Eur. J. Hum. Genet..

[96] Van den Veyver I.B. (2002). Genetic effects of methylation diets. Annu. Rev. Nutr..

[97] Burwell R. (2003). G: Aetiology of idiopathic scoliosis: Current concepts. Pediatr. Rehabil..

[98] Burwell R.G., Dangerfield P.H., Sawatzky B.J. (2004). Hypotheses on the pathogenesis of adolescent idiopathic scoliosis (AIS): A neurodevelopmental concept involving neuronal lipid peroidation and possible prevention by diet. International Research Society of Spinal Deformities Symposium.

[99] Bjornsson H.T., Fallin M.D., Feinberg A.P. (2004). An integrated epigenetic and genetic approach to common human disease. Trends Genet..

[100] Feinberg A.P. (2007). Phenotypic plasticity and the epigenetics of human disease. Nature.

[101] Feinberg A.P. (2008). Epigenetics at the epicenter of modern medicine. JAMA.

[102] Greene N.D., Stanier P., Moore G.E. (2011). The emerging role of epigenetic mechanisms in the etiology of neural tube defects. Epigenetics.

[103] Ford D., Ions L.J., Alatawi F., Wakeling L.A. (2011). The potential role of epigenetic responses to diet in ageing. Proc. Nutr. Soc..

[104] Francis R.C. (2011). A grandmother effect. Epigenetics, the Ultimate Mystery of Inheritance.

[105] Hung V.W.Y., Qin L., Cheung C.S.K., Lam T.P., Ng B.K.W., Tse Y.K., Go X., Lee K.M., Cheng J.C.Y. (2005). Osteopenia: A new prognostic factor of curve progression in adolescent idiopathic scoliosis. J. Bone Jt. Surg. Am..

[106] Lam T.P., Hung V.W., Yeung H.Y., Tse Y.K., Chu W.C., Ng B.K., Lee K.M., Qin L., Cheng J.C. (2011). Abnormal bone quality in adolescent idiopathic scoliosis: A case-control study on 635 subjects and 269 normal controls with bone densitometry and quantitative ultrasound. Spine.

[107] Bagnall K.M., Burwell R.G., Dangerfield P.H., Lowe T.G., Margulies J.Y. (2000). Ligaments and muscles in adolescent idiopathic scoliosis. Etiology of Adolescent Idiopathic Scoliosis: Current Trends and Relevance to New Treatment Approaches, State of the Art Reviews: Spine.

[108] McMaster M., Lee A.J., Burwell R.G., Sawatzky B.J. (2004). Physical activities of patients with adolescent idiopathic scoliosis (AIS) compared with a control group: Implications for etiology and possible prevention. International Research Society of Spinal Deformities Symposium.

[109] McMaster M., Lee A.J., Burwell R.G. (2006). Physical activities of patients with adolescent idiopathic scoliosis (AIS) compared with a control group: Implications for etiology and possible prevention [abstract]. J. Bone Jt. Surg. Br..

[110] Grivas T.B., Vasiliadis E., Mouzakis V., Mihas C., Koufopoulos G. (2006). Association between adolescent idiopathic scoliosis prevalence and age at menarche in different geographic latitudes. Scoliosis.

[111] Cárcamo M., Espinoza P., Rodas M., Urrejola Ó., Bettany-Saltikov J., Grivas T.B. (2023). Prevalence, risk of progression and quality of life assessment in adolescents undergoing school screening for adolescent idopathic scoliosis. Andes Pediatr..

[112] De George F.V., Fisher R.L. (1967). Idiopathic scoliosis: Genetic and environmental aspects. J. Med. Genet..

[113] James J.I.P., Wynne-Davies R., Apley A.G. (1969). Genetic factors in Orthopaedics. Recent Advances in Orthopaedics.

[114] Ryan M.D., Nachemson A. (1987). Thoracic adolescent idiopathic scoliosis: Perinatal and environmental aspects in a Swedish population and their relationship to curve severity. J. Pediatr. Orthop..

[115] Heijmans B.T., Tobi E.W., Lumey L.H., Slagboom P.E. (2009). The epigenome: Archive of the prenatal environment. Epigenetics.

[116] Grivas T.B., Kasartzian A., Mazioti C., Mihas C., Aggouris C., Triantafyllopoulos G., Dimitrakos N., Katsoulis I. (2012). Study of back trunk asymmetry in children from three ethnic groups and correlation with their handedness. Scoliosis.

[117] Grivas T.B., Mihas C., Mazioti C., Zisis N., Sakellaropoulou S., Akriotis A., Burwell R.G. (2012). Maternal age at birth: Does it dictate the epigenotypic expression of the trunkal asymmetry of a child?. Stud. Health Technol. Inform..

[118] McMaster M., Lee A.J., Burwell R.G. (2006). Indoor heated swimming pools: Vulnerability of some infants to develop spinal asymmetries years later. Stud. Health Technol. Inform..

[119] Goldberg C.J., Burwell R.G., Dangerfield P.H., Lowe T.G., Margulies J.Y. (2000). Symmetry control. Etiology of Adolescent Idiopathic Scoliosis: Current Trends and Relevance to New Treatment Approaches, State of the Art Reviews: Spine.

[120] Gluckman P.D., Hanson M.A., Gluckman P., Hanson M. (2006). The developmental origins of health and disease: An overview. Developmental Mechanisms of Health and Disease.

[121] Gluckman P.D., Hanson M.A., Beedle A.S., Buklijas T., Low F.M., Hallgrimsson B., Hall B.K. (2011). Epigenetics of human disease. Epigenetics Linking Genotype and Phenotype in Development and Evolution.

[122] Grunstein J.B., Grivas T.B. (2023). The aetiology of adolescent idiopathic scoliosis: Is sleep position the missing link? The nighttime perfect storm hypothesis. Int. J. Adv. Res..

[123] Viroli G., Ruffilli A., Barile F., Manzetti M., Traversari M., Faldini C. (2024). Pedicle Dysplasia in Proximal Thoracic Adolescent Idiopathic Scoliosis Curves: What are We Missing and What are its Possible Surgical Implications? An Observational Retrospective Study on 104 Patients. Glob. Spine J..

[124] Mehta M.H. (1972). 1972. The rib-vertebra angle in the early diagnosis between resolving and progressive infantile scoliosis. J. Surg. Bone Jt. Br..

[125] Dansereau J., Stokes I.A.F., Moreland M.S., Stokes I.A.F., Pekelsky J.R., Moreland M.S. (1987). Radiographic reconstruction of 3D human rib cage. Surface Topography and Spinal Deformity.

[126] Knott P., Liu X.C. (2021). Eliminating 2D spinal assessments and embracing 3D and 4D: Clinical application of surface topography. Stud. Health Technol. Inform..

[127] Lee T.T., Lai K.K., Cheng J.C., Castelein R.M., Lam T.P., Zheng Y.P. (2021). Investigation of the Phenomenon of Coronal-Sagittal Curvature Coupling on Curve Progression: An Exploratory Study using 3-D Ultrasound. Ultrasound Med. Biol..

[128] Plourde F., Cheriet F., Dansereau J. (2012). Semiautomatic detection of scoliotic rib borders from posteroanterior chest radiographs. IEEE Trans. Biomed. Eng..

[129] Banerjee S., Huang Z., Lyu J., Leung F.H.F., Lee T., Yang D., Zheng Y., McAviney J., Ling S.H. (2024). Automatic Assessment of Ultrasound Curvature Angle for Scoliosis Detection Using 3-D Ultrasound Volume Projection Imaging. Ultrasound Med. Biol..

[130] Cobb J. (1948). Outline for the study of scoliosis. Instr. Course Lect..

[131] Perdriolle R.J., Vidal J. (1981). Etudie de la courbure scoliotique. Importance de l’extension et de la rotation vertebrale. Rev. Chirurie Orthop..

[132] Perdriolle R.J., Vidal J. (1985). Thoracic idiopathic scoliosis curve evolution and prognosis. Spine.

[133] Perdriolle R.J., Vidal J., Bechetti S., Alquier P. Torsion: The essential factor for progression in idiopathic scoliosis. Proceedings of the Combined Meeting of Scoliosis Research Society and European Spinal Deformities Society.

[134] Nash C.L., Moe J.H. (1969). A study of vertebral rotation. J. Bone Jt. Surg Am..

